# Surgical Camouflage as a Solution for Complete Decompensating Failures: An Interesting Experience With Class III Case Scenarios

**DOI:** 10.7759/cureus.25926

**Published:** 2022-06-14

**Authors:** Ratna Parameswaran, Dharshini Ganapathi, Balaji Rajkumar, Anantanarayanan Parameswaran

**Affiliations:** 1 Orthodontics and Dentofacial Orthopedics, Meenakshi Ammal Dental College, Chennai, IND; 2 Oral and Maxillofacial Surgery, Meenakshi Ammal Dental College, Chennai, IND

**Keywords:** soft tissue aesthetics, class iii malocclusion, camouflage, sub apical osteotomy, orthognathic surgery

## Abstract

Dental compensations are an integral part of skeletal malocclusions. Failure to achieve optimal decompensation may lead to compromised surgical movements, thereby resulting in sub-optimal occlusion and soft tissue profile. Hence the mandibular subapical osteotomy was chosen as a minimalistic surgical alternative to the traditional combination of Le Fort 1 and bilateral sagittal split osteotomy. The choice was made by prioritizing soft tissue considerations, which offered the probability of a better post-treatment outcome. This paper highlights two such challenging case scenarios where the surgical plan was modified in accordance with the soft tissue as the primary objective.

## Introduction

Dental compensations are an integral part of skeletal malocclusions. Patients require a variable amount of dentoalveolar decompensation in all three planes of space before orthognathic surgery. This involves the correction of axial inclination of the anterior teeth to the respective skeletal bases aided by fixed orthodontic appliances.

Decompensations are achieved better in the mandible than in the maxilla [1]. In some clinical situations, such as cases with severe crowding and the presence of severely proclined or retroclined incisors [2], complete decompensation is not possible.

Mandibular anterior retroclination and maxillary anterior proclination are the classic dental features observed in skeletal class III malocclusion [1]. Hence pre-surgical orthodontic decompensation includes proclination of the lower and retroclination of the upper anteriors, which are achieved by using a fixed orthodontic appliance. In most situations, the maxillary arch might require extraction for decompensation, particularly when bi-jaw surgery is warranted for achieving a better surgical correction. The decompensations need to be planned with caution to avoid excessive retroclination of the maxillary incisors or excessive proclination of the mandibular incisors beyond the chin point [3]. The efficiency of this pre-surgical orthodontic treatment plays a significant role in deciding the magnitude of surgical movements.

Troy et al. have found that the proclination correction of incisors during decompensation could be achieved only by 50% in class III patients [1]. Other studies also showed the persistence of retroclined lower incisors and proclined upper incisors at the end of pre-surgical orthodontics. General factors like inadequate labial bone lack periodontal support [4] in cases with previous mandibular extraction and crowding, leading to inadequate decompensation [5].

Failure to achieve optimal decompensation may lead to compromised surgical movements, thereby resulting in sub-optimal occlusion and soft tissue profile. Hence, the mandibular subapical osteotomy was chosen as a minimalistic surgical alternative to the traditional combination of LeFort 1 and bilateral sagittal split osteotomy. The choice was made prioritising soft tissue considerations, which offered the probability of a better post treatment outcome.

This paper highlights two such challenging case scenarios of skeletal class III malocclusion where the surgical plan was modified in accordance with the soft tissue considerations of the patients.

## Case presentation

Case 1

A 19-year-old male patient with a complaint of speech problems and irregularly placed upper front teeth revealed a concave profile with anterior divergence, average lower facial height, and shallow mentolabial sulcus. Soft tissue examination revealed a thin drape which accentuated the skeletal deformity. 

**Figure 1:**
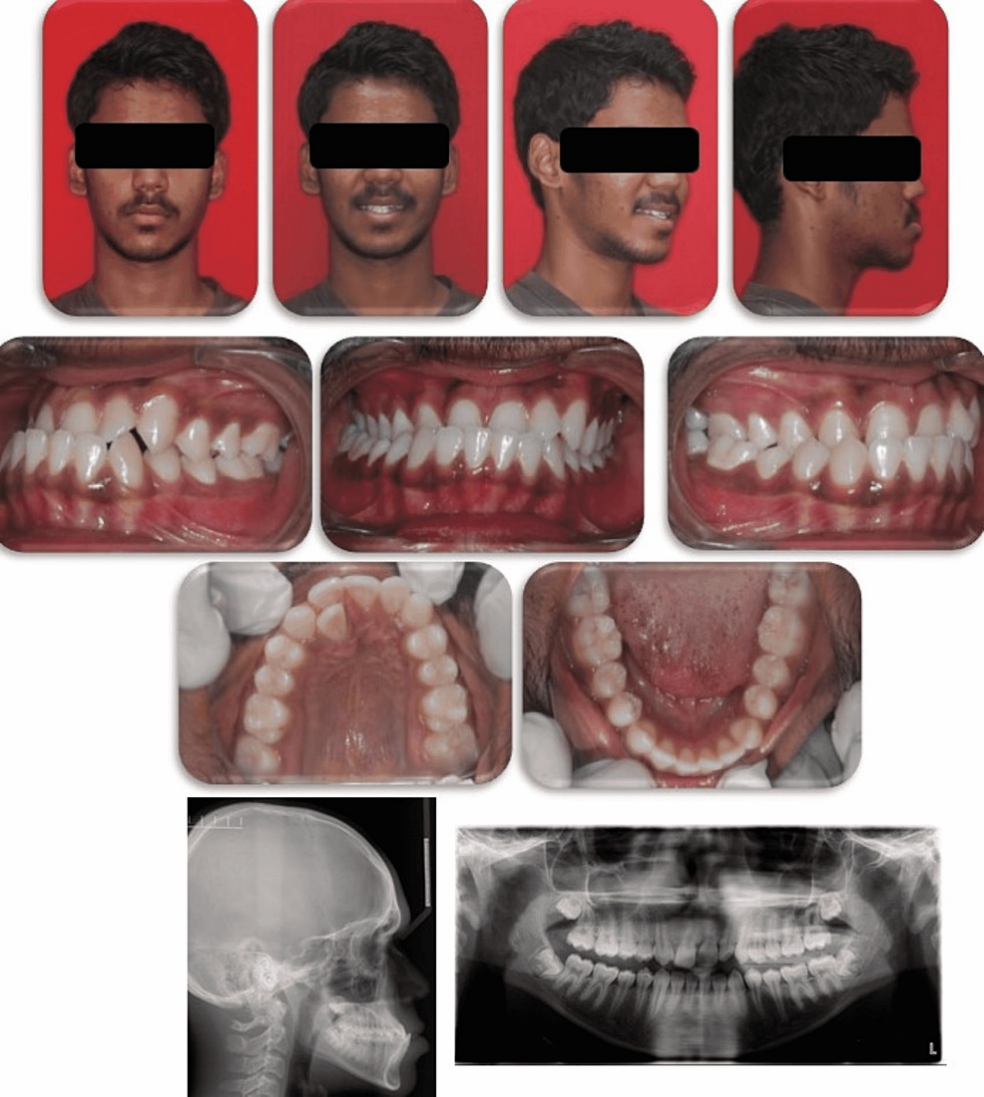
Pre-treatment intraoral and extraoral photographs (lateral cephalogram and orthopantomogram)

Intraoral examination revealed unilateral crossbite on the right side with class III molar relation on both sides and a reverse overjet of 2 mm. The maxillary midline was shifted to the right by 4 mm due to the lingually erupted right maxillary lateral incisor. The maxillary occlusal view showed a collapse in the maxillary arch, which contributed to the unilateral crossbite. The mandibular arch showed accentuated curve of spee (Figure 1). Functional examination revealed functional deviation towards the right side. 

Cephalometric findings confirmed the skeletal class III malocclusion owing to a retrognathic maxilla and a prognathic mandible (SNA=79°; SNB=85°; ANB=-6) on an average mandibular plane angle. Proclined upper and lower anteriors were evident (1 to NA=38°, 1 to NB=27°). Orthopantomogram (OPG) revealed no evidence of bony pathology and the presence of a third molar tooth bud. Poster anterior (PA) cephalogram revealed a mild mandibular shift towards the right side (Figure 1).

The model analysis revealed arch length-tooth material discrepancy and reduced intermolar intercanine and interpremolar width in the upper arch. 

Treatment Plan 

**Figure 2:**
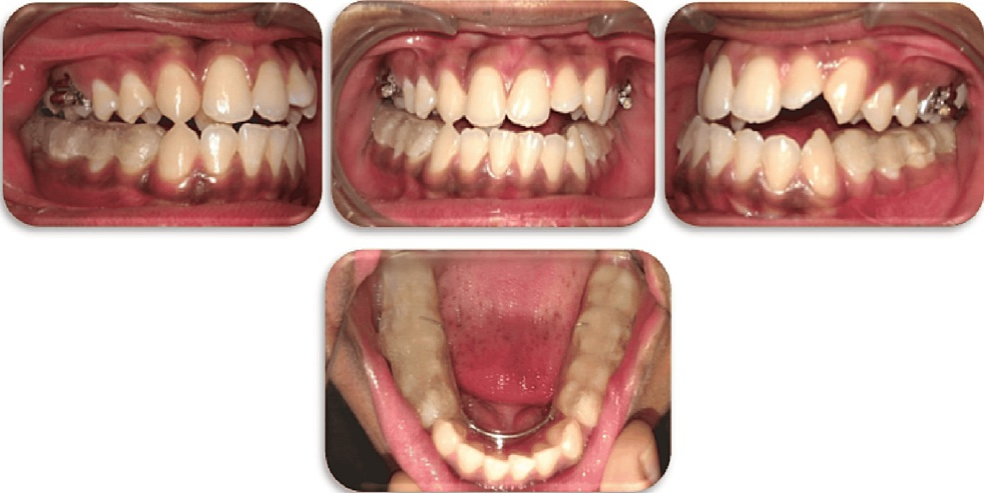
Lower posterior bite plane

The first step involved the correction of the functional deviation with a posterior bite plane (Figure 2).

**Figure 3:**
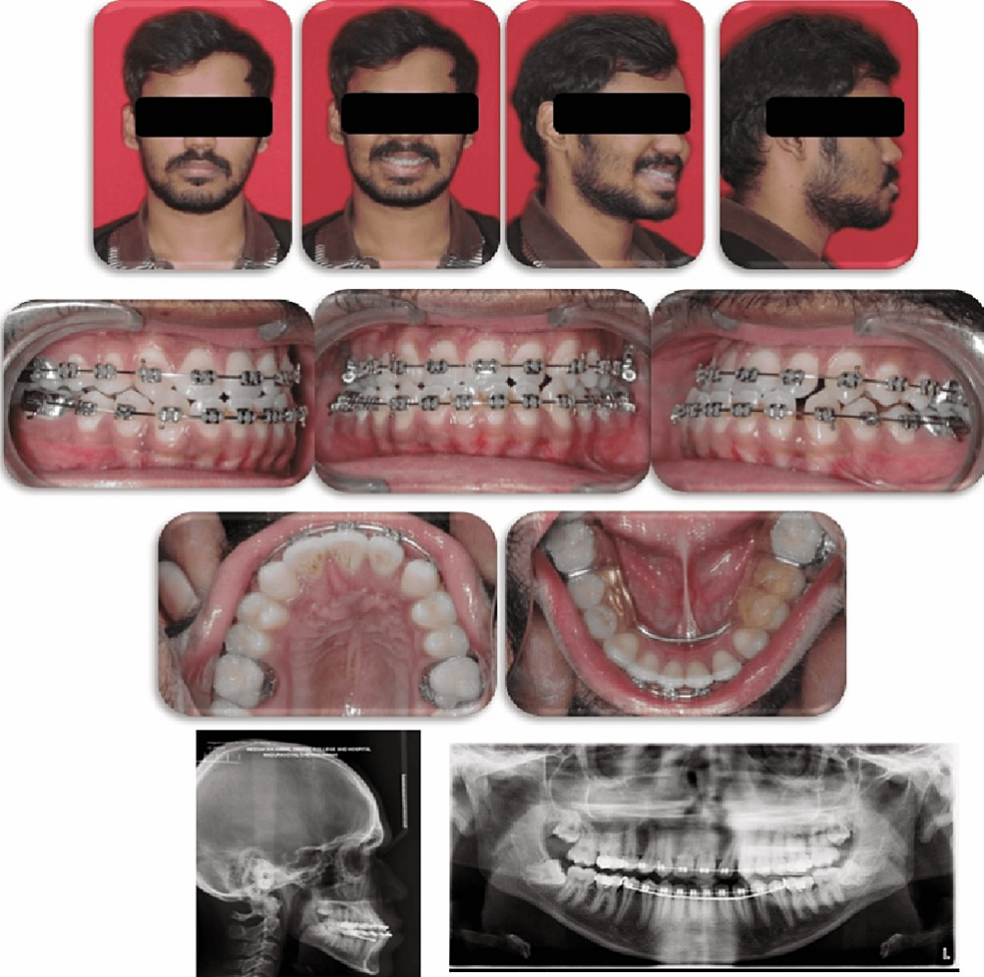
Pre-surgical extraoral and intraoral photographs (lateral cephalogram and orthopantomogram)

The next step was to address the deficient maxilla and prognathic mandible. Partial decompensation involved extraction of 12 and 24 to create the reverse overjet necessary to plan the surgical movements. As the lingually erupted, the right maxillary lateral incisor was not amenable for ideal arch alignment; it was opted for extraction instead of the conventional option of 14 extraction. Pre-surgical orthodontics was commenced using a fixed orthodontic appliance 0.022 MBT system (Ormco, Brea, California). The patient was lost to follow-up subsequently and reported back after four years of abstinence from treatment. Reassessment of the patient revealed profound changes in this clinical status owing to growth. The soft tissue drape continued to remain concave but changed concerning the thickness. Intraoral examination showed proclined maxillary and mandibular teeth, right side in crossbite, and considerable maxillary midline shift towards the right (Figure 3).

At this juncture with the class I molar relationship bilaterally and a minimal reverse overjet of 2 mm, it was evident that the dental decompensations would be insufficient to perform a bi-jaw surgical movement. This led to reassessing the surgical treatment plan. Hence, surgical predictions were carried out to consider the best possible treatment plan. 

**Figure 4:**
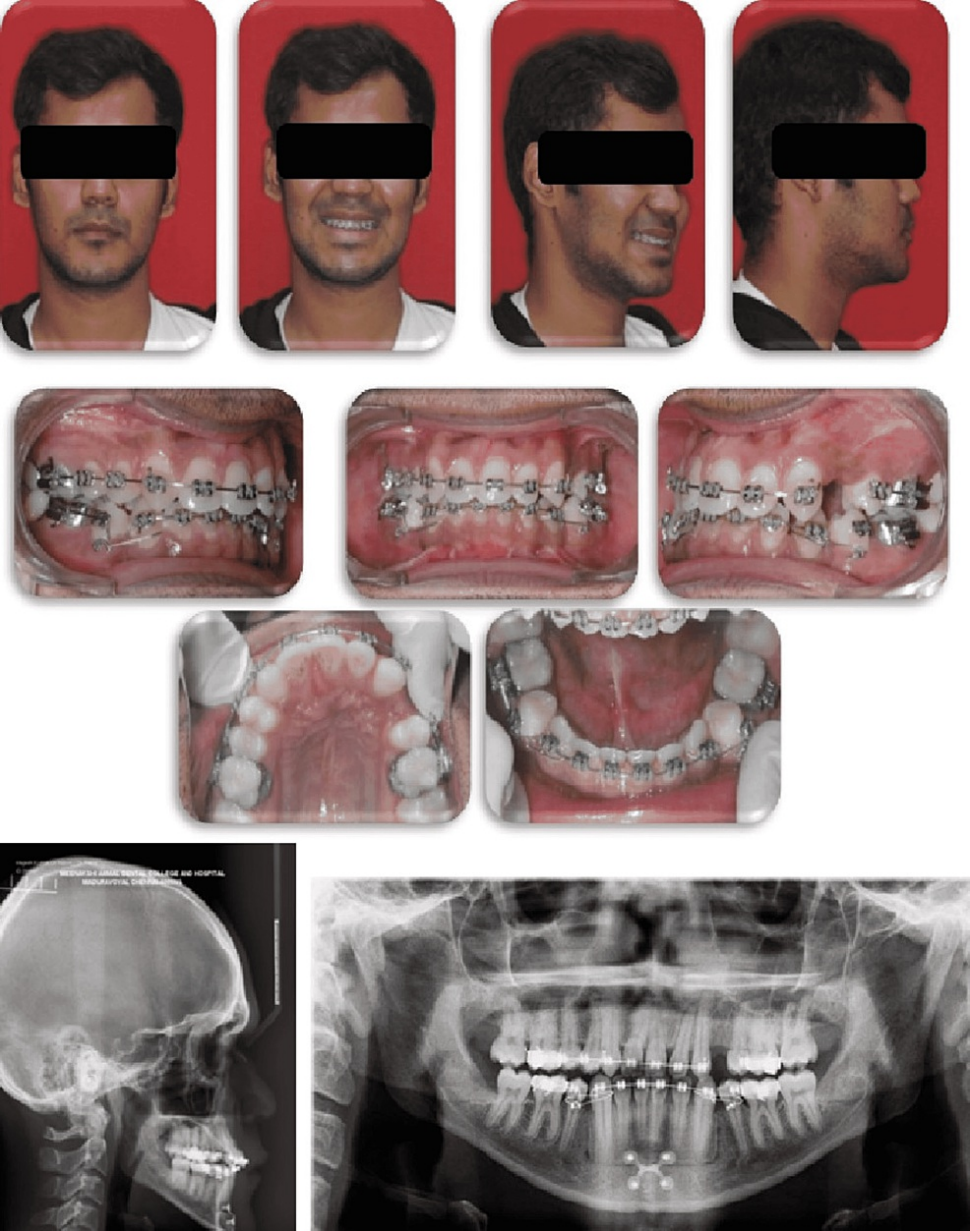
Post-surgery extraoral and intraoral photographs (lateral cephalogram and orthopantomogram)

Assessment of both hard and soft tissue single jaw surgical prediction using software (FACAD, Ilexis AB, Sweden) was performed. BSSO mandibular set-back prediction of 6 mm revealed a skeletal class II profile due to the retrusive chin. Maxillary advancement prediction of 6 mm revealed a bimaxillary protrusive profile owing to the lack of complete decompensation in the upper incisor. Both the above surgical predictions resulted in unpleasant profiles. Hence, the prediction was performed for a segmental mandibular procedure subapical osteotomy, which resulted in an ideal soft tissue profile, as it showed apical base retraction and minimum chin projection, which resulted in good mentolabial sulcus. Subsequent to the prediction, extraction of 24 was planned to correct the dental midline in the maxilla with a quad helix to enable expansion to tackle the crossbite. Extraction of 12 in the maxilla was performed during surgery. Extraction of 34 and 44 in the mandible was also performed intraoperatively to bring about the bodily retraction of the anterior mandibular segment and achieve ideal overbite and jet (Figure 4).

Post-surgically, the extraoral results revealed the fulfillment of all surgical objectives. Intraorally, the resultant occlusion showed class I molar relationship and maxillary right first premolar occluding with lower right canine since 13 replaced 12 and 14 replaced 13. Occlusion on the left showed canine in class II relation and extraction space which was closed. 

The orthodontic retraction phase began immediately within a week after surgery using a frictionless mechanism. The loops were fabricated using 17*25 SS archwire and activated monthly. Class III elastics and settling elastics were implemented for final settling. 

Treatment Results

**Figure 5:**
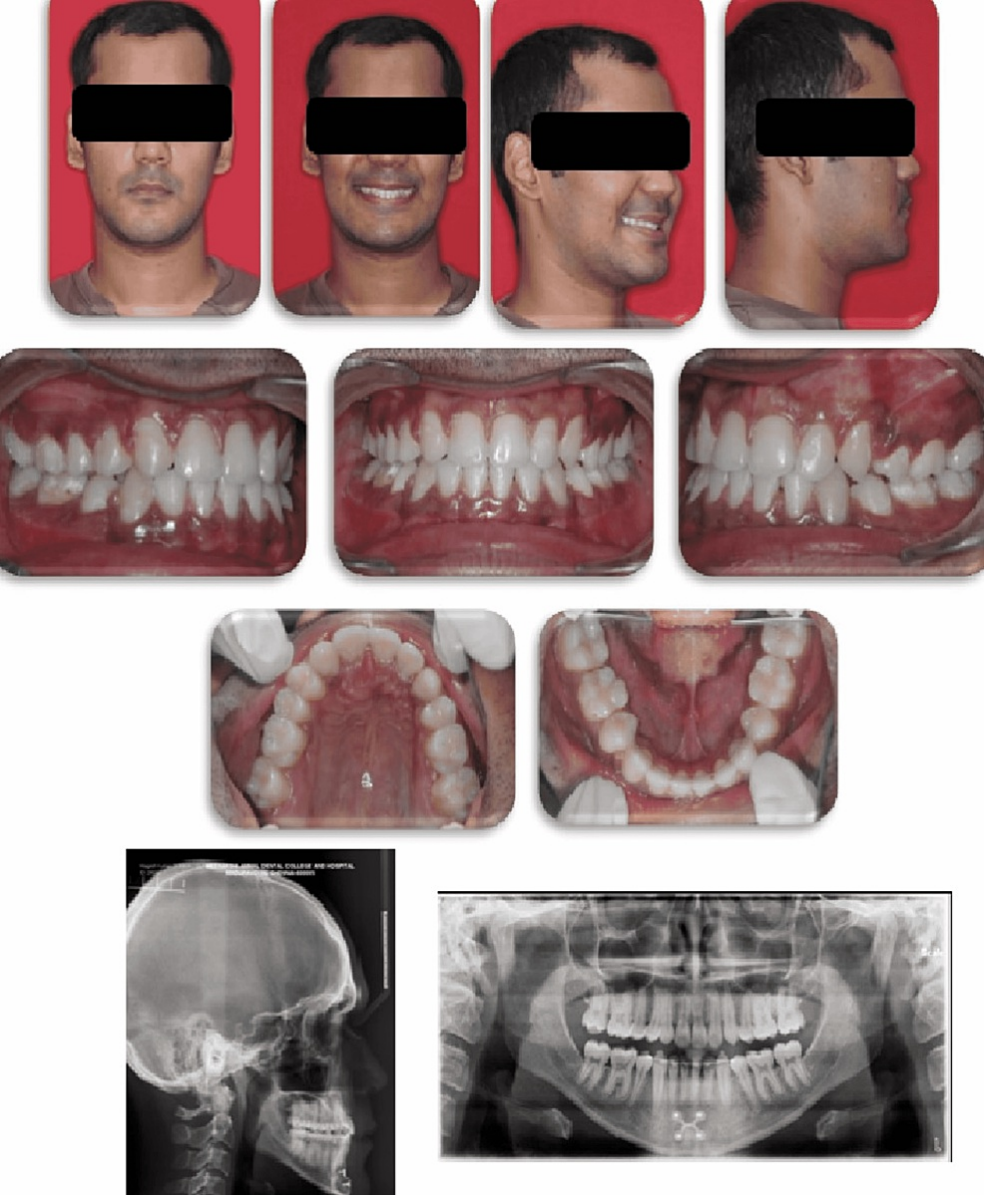
Post-treatment extraoral and intraoral photographs (lateral cephalogram and orthopantomogram)

The fixed appliance was debonded after four months. Overall treatment time was prolonged owing to the patient's failure to comply with the treatment sequence. The treatment was completed with an orthognathic skeletal relationship and a straight pleasing profile extraorally. Intraorally class I molar relationship was achieved bilaterally with ideal overjet and overbite with good occlusion (Figure 5).

Case 2

A 20-year-old female patient reported to us with a complaint of protruding lower jaw and irregular teeth. Clinical features included a concave profile with anterior divergence, severe malar deficiency, and average lower facial height with shallow mentolabial sulcus. 

**Figure 6:**
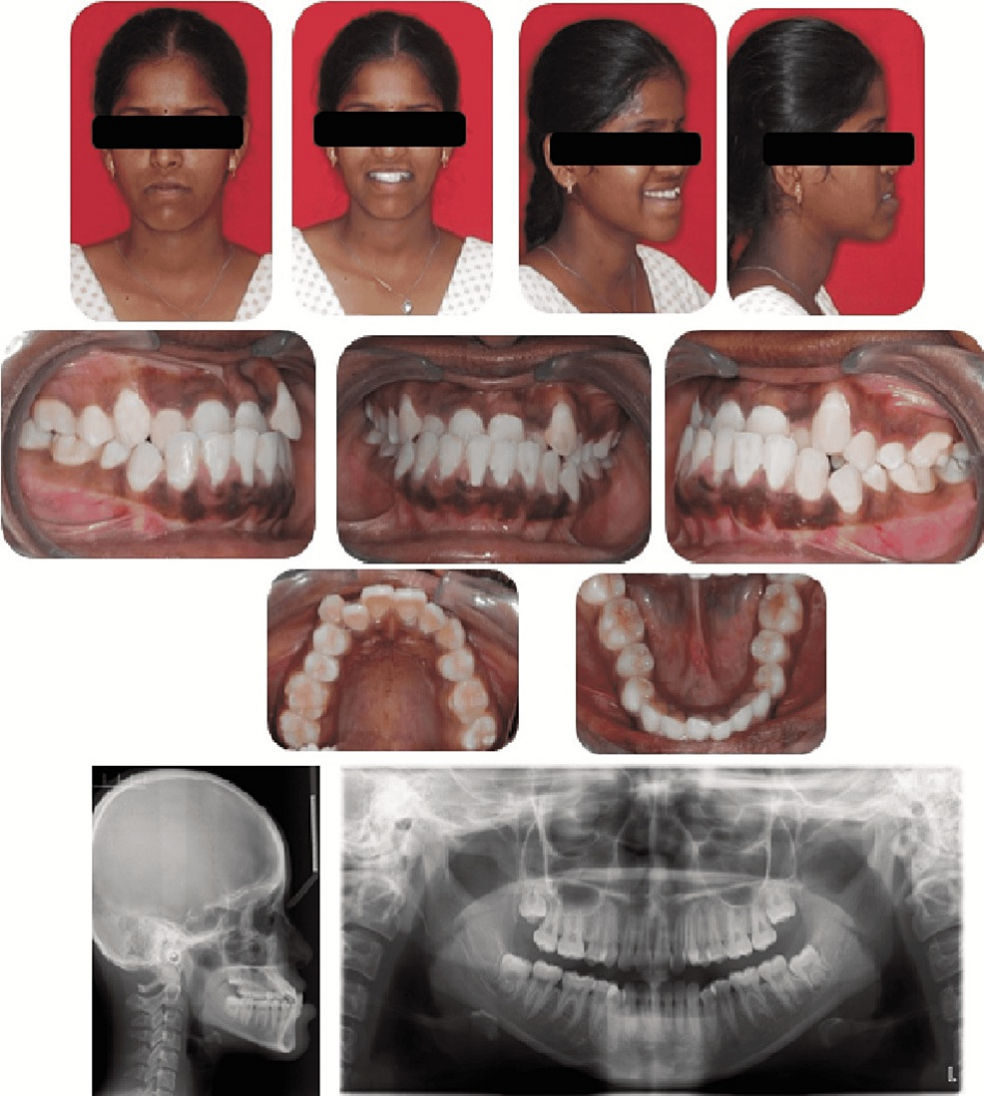
Pre-treatment intraoral and extraoral photographs (lateral cephalogram and orthopantomogram)

Intraoral examination revealed class III molar with class III canine relationship on the right side owing to congenitally missing premolar and class I molar with class II canine relationship on the left side. The anteriors were in a class III relationship exhibiting a reverse overjet of 3 mm with severe proclination of the maxillary anteriors and crowding. Maxillary midline was deviated to the right side by 4 mm. Cephalometric findings revealed a skeletal class III relationship with a retrognathic maxilla, a prognathic mandible (SNA=78°, SNB=84°, ANB=-6), and an average mandibular plane angle. Proclined maxillary and mandibular anteriors were present (1 to NA=35°,1 to NB=29°). Soft tissue analysis showed an acute nasolabial angle, thin upper lip projection increased maxillary incisor exposure, and everted lower lip. The model analysis revealed severe arch length-tooth material discrepancy. OPG revealed no bony pathologies and healthy periodontal status (Figure 6).

Treatment Plan

**Figure 7:**
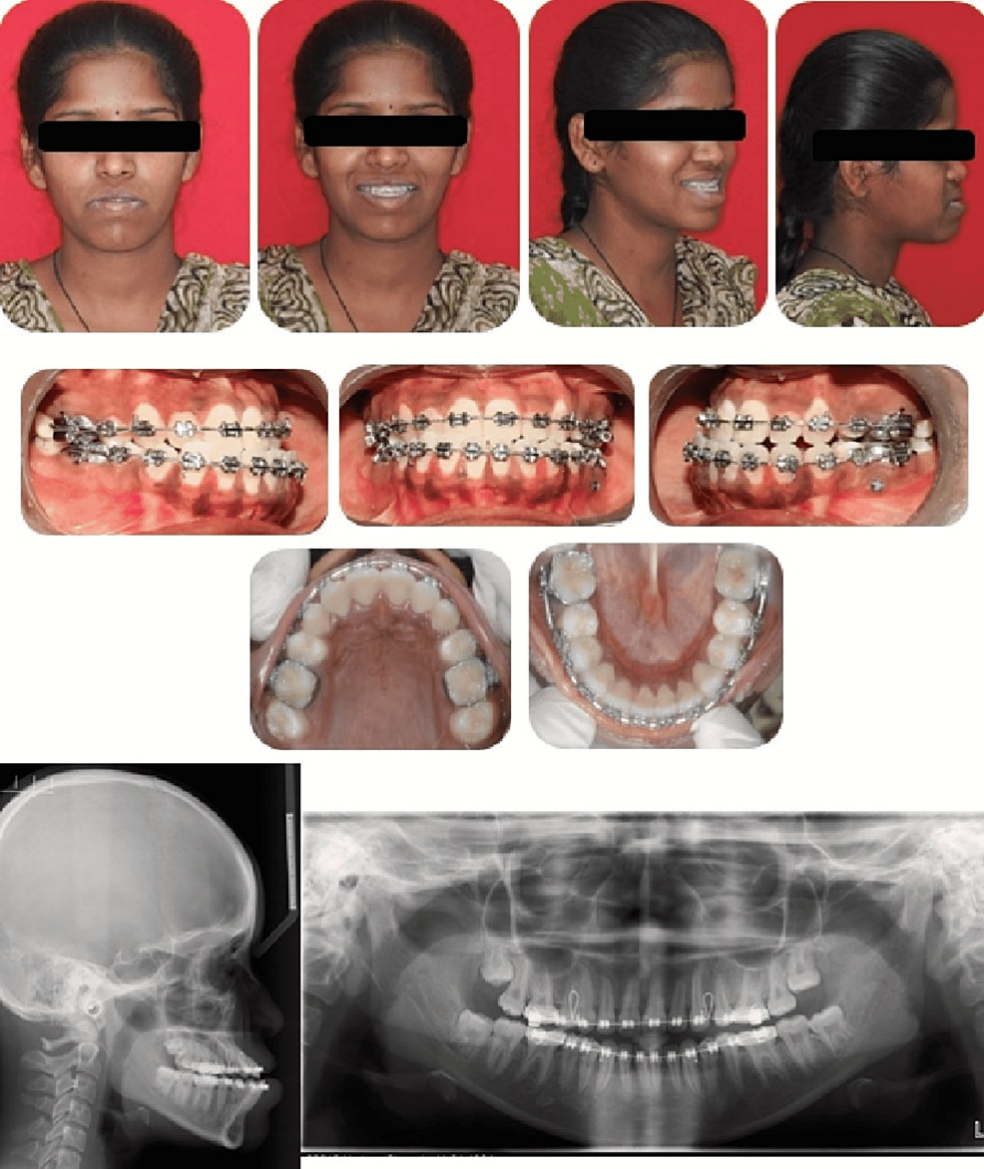
Pre-surgical extraoral and intraoral photographs (lateral cephalogram and orthopantomogram)

The ideal treatment plan was to address both the deficient maxilla and prognathic mandible. Hence the pre-surgical orthodontics involved partial decompensation by extracting unilateral left maxillary first premolar to correct midline shift and proclining the mandibular incisors to achieve adequate reverse overjet for performing a bi-jaw procedure. After the decompensation procedure, the profile was concave. The intraoral features showed class I molar with canine class III relationship on the right side and class II molar with class III canine relationship on the left side. A mini implant was used for augmenting anchorage on the mandibular left quadrant. Maxillary left molar exhibited anchor loss which resulted in the asymmetric molar relationship. The anteriors showed a residual reverse overjet of 2 mm. This incomplete decompensation in the maxillary arch was because of the increased utilization of the extraction spaces for addressing the midline shift and de-crowding over the necessary decompensation. The cephalometric findings, post-decompensation showed a class III skeletal base with proclined maxillary and mandibular incisors (Figure 7).

**Figure 8:**
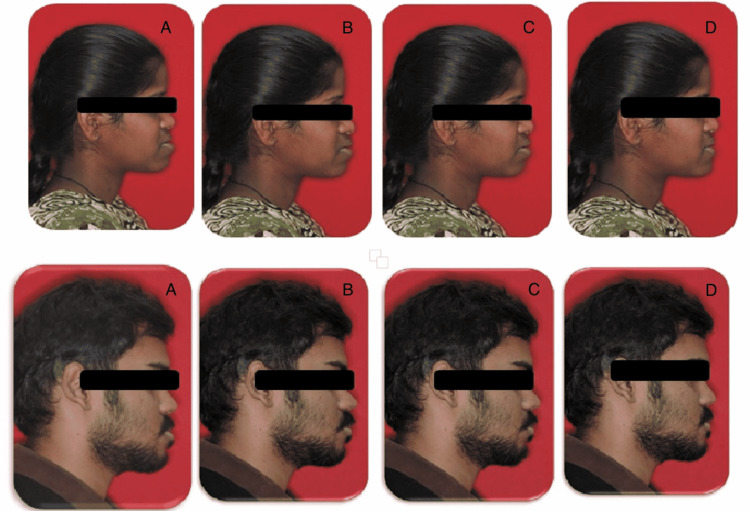
Soft tissue prediction a) pre-surgical; b) maxillary advancement; c) bilateral sagittal split osteotomy (BSSO); d) lower subapical osteotomy

Hence, at the end of decompensation, the reverse overjet was inadequate, and molars relation was not in an ideal decompensated position. The features presented a similar clinical challenge as the previous patient. Similar steps toward predictions and treatment planning were performed (Figure 8).

The patient was treated with mandibular anterior segmental osteotomy and malar grafts to address the malar deficiency. 

**Figure 9:**
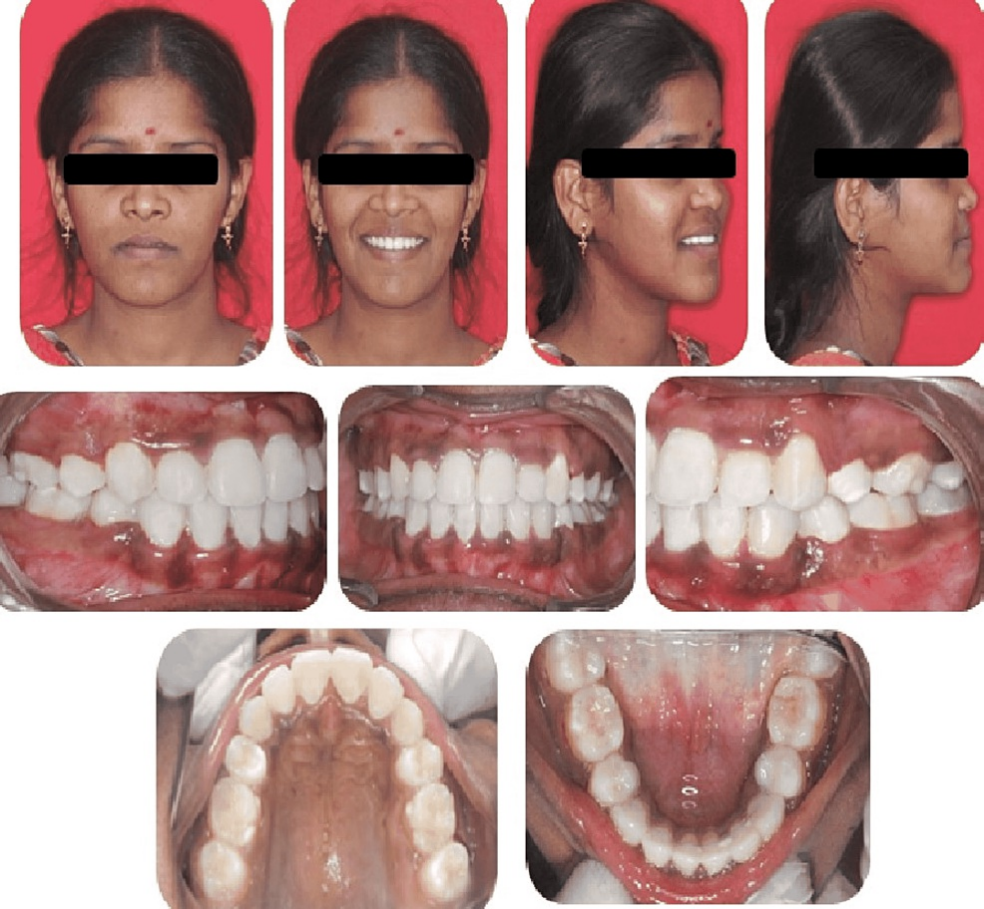
Post-treatment extraoral and intraoral photographs (lateral cephalogram and orthopantomogram)

Post-surgical orthodontics was completed with the correction of molar relationship, torquing the maxillary anteriors, and occlusal settling. Debonding was performed after five months with an overall treatment duration of two years. Post-treatment results showed a balanced soft tissue profile, adequate chin projection, and good cheekbone contour. Intraorally a stable class I relationship in canine and molars were achieved (Figure 9).

## Discussion

This paper highlights the use of surgery as a camouflage option in patients where the pre-surgical decompensation was inadequate to perform conventional surgeries. The conventional orthodontic preparation before surgery is comprised of substantial axial inclination correction of the incisors, thereby relieving the dental compensations. In patients who presented both severities in crowding and proclination, the decompensation leads to inadequate reverse overjet, which forced the clinician to reconsider the surgical options in skeletal class III malocclusion.

In both clinical scenarios, the skeletal deformity was similar. The intraoral features exhibited diversity in the molar and canine relationship. Subsequent to the decompensation, both the patients displayed comparable molar and incisor relationship clinically and cephalometrically. Hence, the plan in both the cases had to be modified to mandibular anterior segmental osteotomy from the bi-jaw conventional surgery bearing in mind the patient's post-treatment soft tissue drape, thereby achieving an ideal aesthetic profile.

The mandibular anterior segmental osteotomy was originally designed to correct alveolar protrusion by retro positioning the osteotomized segment. This procedure offers the advantage of correcting the mandibular incisor position by translating the segment, visibly creating chin projection without performing genioplasty. The total treatment time and extent of orthodontic movements and the biological risks were significantly reduced, not only because of axial dentoalveolar relocation but also due to improved orthodontic efficiency. This also facilitated a favorable soft tissue tone after skeletal base correction and transient demineralization of the operated bone due to the regional acceleratory phenomenon (RAP) [6]. The potential risk of inadequate blood supply is minimized by performing a minimally invasive buccal approach, thereby maintaining the blood supply through the intact periosteal and muscle attachments on the lingual aspect and basal border [7].

The setback of the dental alveolus in the mandibular anterior region contributed to the decreased ANB angle, increased Wits appraisal with the movement, and remodeling of the chin point. The improvement in the Holdaway angle demonstrated the consequent adaptation of the soft tissue [7]. 

The surgical movements had an effective impact on the soft tissue. Predicting the post-surgical soft tissue profile enabled the orthodontist to decide on the surgical procedure as well as inspired the patients for treatment acceptance. Soft tissue consideration played a major role in dental decompensation as well as in orthognathic surgical planning. The final position of the teeth over the basal bone and the soft tissue profile acted as the decision-making criteria for planning the orthognathic surgical procedure [8]. They also influenced the magnitude of movements to be achieved during the time of surgery, as changes were evident in the overlying soft tissue. Hence, quantitative changes in the soft tissue during surgery had to be planned. In both of the above clinical scenarios, multiple surgical predictions were performed, which enabled the clinician to arrive at the modified surgical plan (Figure 9).

The other alternative treatment method would be utilizing the skeletal anchorage system for retraction of the mandibular anterior segment avoiding surgical intervention. Nevertheless, the literature reveals that they bring about good occlusal corrections and do not bring about sufficient skeletal correction, which sequentially doesn't bring about adequate soft tissue change [9]. Bailey et al. stated that the profile rather than occlusion was the main focus of concern for class III patients. Improvement in the profile should play a significant role in evaluating treatment outcomes [10].

## Conclusions

In patients undergoing orthognathic surgery, decompensation often involves therapeutic extractions. When extraoral soft tissue thickness masks the underlying severe skeletal deformity, extractions, and surgery planning need to be modified. The conventional surgical plan, which generally involves bi-jaw procedures, is modified with a less invasive plan providing the patient with optimal aesthetic and functional outcomes. Minimally aggressive surgeries such as subapical osteotomy should be considered as an alternative option for conventional bi-jaw surgeries where failure to accomplish complete pre-surgical decompensation in skeletal class III malocclusion. Hence, these case series highlight the importance of soft tissue-based diagnosis and minimal surgical intervention for an effective clinical outcome.

## References

[1] McNeil C, McIntyre GT, Laverick S (2014). How much incisor decompensation is achieved prior to orthognathic surgery?. J Clin Exp Dent.

[2] Potts B, Shanker S, Fields HW, Vig KW, Beck FM (2009). Dental and skeletal changes associated with class II surgical-orthodontic treatment. Am J Orthod Dentofacial Orthop.

[3] Klein KP, Kaban LB, Masoud MI (2020). Orthognathic surgery and orthodontics: inadequate planning leading to complications or unfavorable results. Oral Maxillofac Surg Clin North Am.

[4] Handelman CS (1996). The anterior alveolus: Its importance in limiting orthodontic treatment and its influence on the occurrence of iatrogenic sequelae. Angle Orthod.

[5] Ahn HW, Lee DY, Park YG, Kim SH, Chung KR, Nelson G (2012). Accelerated decompensation of mandibular incisors in surgical skeletal class III patients by using augmented corticotomy: a preliminary study. Am J Orthod Dentofacial Orthop.

[6] Hernández-Alfaro F, Nieto MJ, Ruiz-Magaz V, Valls-Ontañón A, Méndez-Manjón I, Guijarro-Martínez R (2017). Inferior subapical osteotomy for dentoalveolar decompensation of class III malocclusion in 'surgery-first' and 'surgery-early' orthognathic treatment. Int J Oral Maxillofac Surg.

[7] Rabie AB, Wong RW, Min GU (2008). Treatment in borderline class III malocclusion: orthodontic camouflage (extraction) versus orthognathic surgery. Open Dent J.

[8] Luther F, Morris DO, Hart C (2003). Orthodontic preparation for orthognathic surgery: how long does it take and why? A retrospective study. Br J Oral Maxillofac Surg.

[9] Meyns J, Brasil DM, Mazzi-Chaves JF, Politis C, Jacobs R (2018). The clinical outcome of skeletal anchorage in interceptive treatment (in growing patients) for class III malocclusion. Int J Oral Maxillofac Surg.

[10] Bailey LJ, Haltiwanger LH, Blakey GH, Proffit WR (2001). Who seeks surgical-orthodontic treatment: a current review. Int J Adult Orthodon Orthognath Surg.

